# Supplementary material for: End Sequence Analysis Toolkit (ESAT) expands the extractable information from single-cell RNA-seq data
Source: Genome Res. 2016 Oct;26(10):1397–410. doi: 10.1101/gr.207902.116 (PMC5052061; doi:10.1101/gr.207902.116)
Supplement: Supplemental Material [file supp_gr.207902.116_Supp_Materials.tar.gz › ESAT-esat_v0.1/doc/html/classbroad_1_1pda_1_1samtools_1_1_s_a_m_paired_end_file_reader.html]

ESAT: broad.pda.samtools.SAMPairedEndFileReader Class Reference


ESAT


- Main Page
- Related Pages
- Packages
- Classes
- Files

- Class List
- Class Index
- Class Hierarchy
- Class Members

All Classes Namespaces Files Functions Variables Enumerator Pages

- broad
- pda
- samtools
- SAMPairedEndFileReader

Classes |
Public Member Functions |
Static Public Member Functions |
Static Public Attributes |
List of all members

broad.pda.samtools.SAMPairedEndFileReader Class Reference

Inheritance diagram for broad.pda.samtools.SAMPairedEndFileReader:

[legend]

Collaboration diagram for broad.pda.samtools.SAMPairedEndFileReader:

[legend]

|  |  |
| --- | --- |
| Public Member Functions | |
|  | SAMPairedEndFileReader (final File file) |
|  | |
| CloseableIterator< Alignment > | iterator () |
|  | |
| CloseableIterator< SAMRecord > | getSAMRecordIterator () |
|  | |
| void | close () |
|  | |
| SAMFileReader | getReader () |
|  | |
| CloseableIterator< Alignment > | query (String sequence, int start, int end, boolean contained) |
|  | |
| SAMFileHeader | getHeader () |
|  | |
| Set< String > | getSequenceNames () |
|  | |
| boolean | hasIndex () |
|  | |

|  |  |
| --- | --- |
| Static Public Member Functions | |
| static SAMRecord | convertToPairedEndFragment (SAMRecord rec) |
|  | |

|  |  |
| --- | --- |
| Static Public Attributes | |
| static final int | MAX\_INSERT = 2000 |
|  | |

## Detailed Description

Author
:   engreitz Modeled after the Picard SAMFileReader Currently this class does not just extend the Picard SAMFileReader because that class has a lot more functionality that would be a pain to implement in the paired-end format.

Also it's trying to accommodate iterators of both SAMRecords and Alignments to be compatible with Scripture/IGV. Annoying.

Note: With move to ScriptureV2, this class was replaced with nextgen.core.writers.PairedEndWriter and nextgen.core.readers.PairedEndReader

## Constructor & Destructor Documentation

|  |  |  |  |  |  |
| --- | --- | --- | --- | --- | --- |
| broad.pda.samtools.SAMPairedEndFileReader.SAMPairedEndFileReader | ( | final File | *file* | ) |  |

## Member Function Documentation

|  |  |  |  |  |
| --- | --- | --- | --- | --- |
| void broad.pda.samtools.SAMPairedEndFileReader.close | ( |  | ) |  |

|  |  |  |  |  |  |
| --- | --- | --- | --- | --- | --- |
| static SAMRecord broad.pda.samtools.SAMPairedEndFileReader.convertToPairedEndFragment | ( | SAMRecord | *rec* | ) |  |

static

|  |  |  |  |  |
| --- | --- | --- | --- | --- |
| SAMFileHeader broad.pda.samtools.SAMPairedEndFileReader.getHeader | ( |  | ) |  |

Here is the caller graph for this function:

|  |  |  |  |  |
| --- | --- | --- | --- | --- |
| SAMFileReader broad.pda.samtools.SAMPairedEndFileReader.getReader | ( |  | ) |  |

|  |  |  |  |  |
| --- | --- | --- | --- | --- |
| CloseableIterator<SAMRecord> broad.pda.samtools.SAMPairedEndFileReader.getSAMRecordIterator | ( |  | ) |  |

|  |  |  |  |  |
| --- | --- | --- | --- | --- |
| Set<String> broad.pda.samtools.SAMPairedEndFileReader.getSequenceNames | ( |  | ) |  |

Here is the call graph for this function:

|  |  |  |  |  |
| --- | --- | --- | --- | --- |
| boolean broad.pda.samtools.SAMPairedEndFileReader.hasIndex | ( |  | ) |  |

|  |  |  |  |  |
| --- | --- | --- | --- | --- |
| CloseableIterator<Alignment> broad.pda.samtools.SAMPairedEndFileReader.iterator | ( |  | ) |  |

|  |  |  |  |
| --- | --- | --- | --- |
| CloseableIterator<Alignment> broad.pda.samtools.SAMPairedEndFileReader.query | ( | String | *sequence*, |
|  |  | int | *start*, |
|  |  | int | *end*, |
|  |  | boolean | *contained* |
|  | ) |  |  |

## Member Data Documentation

final int broad.pda.samtools.SAMPairedEndFileReader.MAX\_INSERT = 2000

static

---

The documentation for this class was generated from the following file:

- java/broad/pda/samtools/SAMPairedEndFileReader.java


---

Generated on Fri Aug 15 2014 15:11:01 for ESAT by  

 1.8.7
